# Analysis of Interarch Tooth Size Relationship in Nepalese Subjects with Normal Occlusion and Malocclusions

**DOI:** 10.1155/2019/2761427

**Published:** 2019-11-18

**Authors:** Rajeev Kumar Mishra, Dashrath Kafle, Rahul Gupta

**Affiliations:** ^1^Kathmandu University School of Medical Sciences, Dhulikhel, Nepal; ^2^M. B. Kedia Dental College and Hospital, Birgunj, Nepal

## Abstract

**Introduction:**

A proportional relationship between the maxillary and mandibular teeth size is required for achieving good finish with proper overjet and overbite postorthodontic treatment. The aims and objectives of this study were to determine the anterior and overall Bolton's ratio in Nepalese population, to compare Bolton's ratio between subjects with normal occlusion, Class I malocclusion, and Class II malocclusion, to compare these results with Bolton's norm, and to determine the frequency of clinically significant (beyond 2 SD) tooth size discrepancy compared to Bolton's norm.

**Materials and Methods:**

The study models of the subjects with normal occlusion and Angle's Class I malocclusion and Class II malocclusion and fulfilling the inclusion criteria were retrieved from department archives. An electronic digital caliper was used to measure mesiodistal tooth size of the maxillary and mandibular teeth anterior to the second molars. The study sample of 120 study models consisted of the normal occlusion group (*n* = 31), Class I malocclusion group (*n* = 47), and Class II malocclusion group (*n* = 42). These measurements were then used to obtain Bolton's ratio in three groups of subjects. Bolton's ratio of study groups was compared with each other and with Bolton's original ratio.

**Results:**

The differences in tooth size ratio of the study groups were not significant statistically, when the groups were compared on the basis of malocclusion or gender. Statistically significant differences were exclusively observed between the study groups and Bolton's original sample for the anterior ratio. The frequency of the clinically significant tooth size ratio discrepancy was lower for the overall ratio (9.1%) compared to the anterior ratio (22.5%).

**Conclusions:**

Bolton's analysis on the Nepalese population sample shows that there was no significant difference observed on the anterior and overall tooth size ratios when these were compared based on Angle's malocclusion classes or gender. The clinically significant anterior tooth size discrepancy was more prevalent than that of the overall ratio.

## 1. Introduction

A proportional relationship between the maxillary and mandibular tooth size is required for achieving good finish with proper overjet and overbite postorthodontic treatment [1]. The absence of tooth size discrepancy had been considered the seventh key to normal occlusion [2]. The presence of tooth size discrepancy should be identified during the initial diagnosis, and treatment planning stage and appropriate mechanism should be applied for resolving the discrepancy.

The effect of tooth size discrepancy on occlusion has been reported since the early years of modern orthodontics. The ideal proportion of the maxillary and mandibular tooth material had been expressed as numerical differences in their size [3, 4], percentage, and the ratios [5–7]. Among these, Bolton's ratio is one of the most commonly used methods to determine interarch tooth size discrepancy in orthodontic patients [1]. However, due to the selection bias in Bolton's study (subjects with an excellent occlusion were selected for his study, while population and gender characteristics of the study sample were unspecified), Bolton's ratio may differ in the subjects with malocclusions and in different population groups [8–12]. The results from the previous studies on the interarch tooth discrepancy based on the malocclusions and in different racial groups are not in concordance. Smith et al. examined the Bolton ratio in three population groups, namely, Black, Spanish, and White, with the conclusion that Bolton's ratio applies to White female only and should not be indiscriminately applied to the White male, Hispanic, and Black population [8]. Johe et al. have reported that when compared with Caucasian and Hispanic patients, African American patients had significantly greater odds of having a clinically significant (±2 SD) anterior ratio discrepancy [9]. In contrast, Endo et al. found no significant difference between the anterior or the overall ratio of Japanese and Bolton's original study group and stated that Bolton's values can be used with confidence in the Japanese orthodontic population [13].

According to Proffit and Ackerman, the tooth size discrepancy of greater than 1.5 mm may jeopardize optimal finishing and hence should be considered in the treatment plan [14], while Othman and Haaradine stated that a 2 mm of required tooth size correction is an appropriate threshold for clinical significance [15]. Many previous studies have accepted a deviation of 2 SD outside Bolton's mean value as clinically significant [12, 13, 15, 16]. However, no evidence has been given regarding the clinical significance of these values, and these values seem to be suggestions [17]. The frequency of clinically significant tooth size discrepancy reported by various studies is highly variable; however, majority of the studies report that the anterior tooth size discrepancy is more frequent than the overall tooth size discrepancy [13, 15–17].

The aims and objectives of this study were to determine the anterior and overall Bolton's ratio in Nepalese population, to compare these ratios between subjects with normal occlusion, Class I malocclusion, and Class II malocclusion, to compare these results with Bolton's norm, and to determine the frequency of clinically significant (beyond 2 SD) tooth size discrepancy compared to Bolton's norm.

## 2. Materials and Methods

The study was carried out at the Department of Orthodontics in a dental college hospital. The study sample was selected from the department archives. The subjects were selected based on following inclusion criteria:

- Normal occlusion group: Angle's Class I molar and canine relationship bilaterally, no history of previous orthodontic treatment, complete dentition up to at least permanent first molars, regular arch form with mild (≤3 mm) or no crowding, normal overjet and overbite(2 mm ± 1 mm), mild (≤2 mm) or no spacing, and absence of large restorations.
- Class I malocclusion group: Angle's Class I malocclusion, no history of previous orthodontic treatment, and complete dentition up to at least permanent first molars.
- Class II malocclusion group: Angle's Class II malocclusion, no history of previous orthodontic treatment, and complete dentition up to at least permanent first molars. Owing to small number of Class II division 2 samples in the archive, only Class II division 1 samples were used for this study.

The Class III malocclusion group was not assessed in the present study because the department archive had records of only five Class III malocclusion cases.

The demographic distribution of these groups is depicted in the Table 1.

The mesiodistal width of anterior and posterior teeth up to permanent first molars were measured in the maxillary and mandibular arch with an electronic digital caliper (range: 0–150 mm, accuracy: ±0.02 mm). The mesurements were made perpendicular to the long axis of tooth. All measurements were made by a single investigator (RKM), and the teeth were measured to the nearest 0.1 mm. The anterior and overall Bolton's ratios were calculated using the Microsoft Excel program.

The following formulae were used to calculate the anterior and overall ratios:

(1) $$\begin{array}{c} \text{anterior ratio}=\frac{\text{sum of mesiodistal width of }33-43}{\text{sum of mesiodistal width of }13-23\;}\times 100\%,\\ \text{overall ratio}=\frac{\text{sum of mesiodistal width of }36-46}{\text{sum of mesiodistal width of }16-26}\times 100\%. \end{array}$$

To assess the error of the method, the mesiodistal width of maxillary teeth of 15 randomly selected subjects were remeasured and the differences in the measurements were analyzed using the paired *t*-test to calculate the systematic error and Dahlberg's formula to assess the casual error. The independent *t*-test was used to analyze the difference between tooth size of males and females. One-way ANOVA was used to assess the Bolton ratio difference between the groups as function of Angle's malocclusion and gender. The one sample *t*-test was used to compare the differences between the groups of the present study and Bolton's original sample. The level of significance was fixed at 5% (*p* ≤ 0.05).

## 3. Results

The causal error assessed by Dahlberg's formula ranged from 0.18 to 0.48. The paired *t*-test revealed that the systematic error was not significant (Table 2). The independent *t*-test showed that there was no difference between tooth size of the male and female subjects (Table 3). One-way ANOVA showed that there was no significant difference in the anterior and overall ratio between the groups when compared on the basis of gender or Angle's malocclusion (Tables 4 and 5). The one sample *t*-test was used to assess the differences between the present study groups and Bolton's original sample. A significant difference, however, limited to the anterior ratio only was observed between the normal occlusion and Class II malocclusion of the present study group and Bolton's original sample (Table 6). The frequency of clinically significant (mean ± 2 SD) discrepancy was higher for the anterior ratio (22.5%) compared to the overall ratio (9.1%). The clinically significant anterior ratio discrepancy was most frequently observed in Class II malocclusion (26.1%), while the overall ratio was more prevalent for the Class I malocclusion sample (12.7%). For the anterior ratio, mandibular excess occurred in all the groups, whereas incidence of mandibular excess was observed in Class I group only (Tables 7 and 8) for the overall ratio.

## 4. Discussion

The aims of the study were to determine Bolton's anterior and overall ratios in Nepalese subjects with normal occlusion and Angle's Classes I and II malocclusions. The Class III subjects were not included in the present study because of limited availability of the records of Class III subjects in the department archives. The prevalence of Class III malocclusion in Nepal is very low as reported in the previous studies. In the Eastern Nepalese population, Sharma JN had reported a prevalence of 3.7% [18], while Acharya et al. [19] had documented prevalence of 0.66%. In the Western Nepal, Baral [20] have reported a prevalence of 4.1%, while Halwai and Gautam [21] have reported it to be 4.5%.

In our study, no significant differences were found between males and females regarding the sum of teeth dimensions and Bolton's anterior and overall ratios. There is conflicting evidence regarding the extent of sexual dimorphism with respect to the tooth size ratio. In our study, no significant differences were found with respect to gender for the anterior ratio and overall ratio. This is in agreement with majority of previously reported findings [12, 22–24], but contrasting findings have been reported by other studies [8, 25]. In the Nepalese subjects, Hong et al. have reported no significant difference between male and female Class I samples for either anterior ratio or overall ratio [26]; however, Jaiswal et al. have reported significant difference between male and female subjects for anterior ratio only [27]. Our findings are similar to that reported by Akyalçin et al. in Turkish population [1], Endo et al. in Japanese population [13], Machado et al. in Portuguese population [28], and Ismail et al. in Sudanese population [29]. However, Smith et al. who studied the Bolton interarch ratio for 3 population groups, namely, Blacks, Hispanics, and Whites, concluded that interarch tooth size relationships are population and gender specific [8]. They have reported significant difference between the genders for the overall ratio but not for the anterior ratio. Mollabashi et al. have also reported a relationship between gender and overall ratio only [25].

In our study, no significant differences were observed with regard to anterior and overall ratios when compared as a function of Angle's malocclusion. Our findings concur with most of the previous studies reported from different population groups [1, 9–13, 22]. However, our findings are in contrast to those reported by some authors. Araujo and Sauoki have reported that the anterior tooth size ratio for Angle's Class III subjects was significantly greater than that of Class I and Class II subjects [16]. Nie and Lin have reported significant difference between malocclusion groups with respect to both anterior and overall ratios [23]. The ratio values were highest for Class III and lowest for Class II groups. Similar findings have been reported by Prasanna et al. in Indian population [30]. In our view, as Class III subjects were not included in our study, a direct comparison of findings of our study with these studies seems inappropriate, but based on our study findings and the literature search, we will like to conclude that the tooth size ratio is independent of malocclusion type.

Statistically significant differences were observed between the normal occlusion and Class II groups of the study when those groups were separately compared with Bolton's original sample for the anterior ratio and also when all the three groups were combined together for comparison. However, no significant differences were observed for the overall ratio. This is similar to the findings reported by Hashim et al. [22] who have found significant difference between Qatari population (malocclusion characteristics unspecified) and Bolton's original sample with respect to anterior ratio only. Shastri et al. who studied tooth size discrepancy in North Indian population have reported that the mean anterior ratio for Angle's Class II subjects was significantly greater compared to Bolton's mean anterior ratio [31]. However, contrasting findings have been reported by Ricci et al. [10] who have reported significant difference with respect to the anterior ratio for Class I malocclusion groups and with respect to the overall ratio for the normal occlusion group.

In our study, the frequency of clinically significant tooth size discrepancy (beyond ± 2 SD of Bolton's mean) was higher for the anterior ratio when compared with the overall ratio. Our findings are in concordance with that reported by the previous studies. Endo et al. have reported that, in Japanese population, the clinically significant anterior tooth size discrepancy was present in 14.4% of subjects, while the prevalence of clinically significant overall ratio was seen in 6.66% [13]. Othman and Harradine have reported prevalence of 17.4% and 5.4% for clinically significant anterior and overall ratios [15]. Cancado et al. in Brazilian population have reported the prevalence of anterior ratio discrepancy in 23.4% subjects and overall ratio discrepancy in 6.5% subjects [32]. The greater prevalence of clinically significant anterior tooth size discrepancy compared to overall ratio discrepancy may be due to greater variations in the size of the anterior teeth.

## 5. Conclusions

1. There was no significant difference between male and female subjects for anterior and overall tooth size ratios
2. There was no significant difference between normal occlusion, Class I, and Class II malocclusion groups with respect to anterior and overall tooth size ratios
3. Statistically significant differences were observed only for anterior tooth size ratio when the study groups were compared with Bolton's original ratio
4. The prevalence of clinically significant anterior tooth size discrepancy was higher than that of the overall ratio

## Figures and Tables

**Table 1:** Group characteristics.

Group	Male	Female	Total	Mean age (years)
Normal occlusion	11	20	31	21.8 ± 2.5
Class I malocclusion	22	25	47	20.2 ± 5.2
Class II malocclusion	18	24	42	19.2 ± 4.5

**Table 2:** Analysis of error of the method using the paired *t*-test and Dahlberg's formula.

Tooth number	First measurement	Second measurement	*p* value	Casual error
11	8.47 ± 0.55	8.32 ± 0.74	0.12	0.38
12	6.86 ± 0.42	6.78 ± 0.57	0.26	0.22
13	7.66 ± 0.45	7.52 ± 0.61	0.23	0.37
14	6.9 ± 0.47	7.0 ± 0.53	0.17	0.27
15	6.33 ± 0.54	6.39 ± 0.68	0.63	0.18
16	9.9 ± 0.48	9.8 ± 0.8	0.41	0.27
21	8.46 ± 0.56	8.38 ± 0.68	0.33	0.21
22	6.8 ± 0.44	6.7 ± 0.41	0.09	0.24
23	7.53 ± 0.46	7.46 ± 0.5	0.33	0.22
24	6.96 ± 0.43	6.87 ± 0.46	0.21	0.26
25	6.31 ± 0.61	6.19 ± 0.67	0.07	0.30
26	9.86 ± 0.53	10.03 ± 0.57	0.14	0.48

**Table 3:** Independent *T*-test for comparison of tooth size between males and females of different groups.

Group	Tooth size	Male (mean ± SD)	Female (mean ± SD)	*p* value
Normal occlusion	Anterior ratio	78.0 ± 1.8	78.2 ± 2.6	0.81
	Overall ratio	91.5 ± 2.1	91.7 ± 1.8	0.85

Class I malocclusion	Anterior ratio	77.0 ± 2.9	78.4 ± 2.3	0.07
	Overall ratio	91.6 ± 2.7	91.2 ± 2.4	0.61

Class II malocclusion	Anterior ratio	78.0 ± 3.1	78.3 ± 2.5	0.74
	Overall ratio	91.5 ± 1.6	91.3 ± 2.3	0.59

**Table 4:** Comparison of tooth size and Bolton's anterior and overall ratios as a function of Angle's malocclusion.

Variable	Normal	Class I	Class II	*p* value
Anterior ratio	78.1 ± 2.4	77.8 ± 2.7	78.2 ± 2.8	0.78
Overall ratio	91.6 ± 1.9	91.3 ± 2.5	91.3 ± 2.2	0.84

**Table 5:** Comparison of Bolton's anterior and overall ratios as a function of sex and Angle's classification.

		Male	*p* value	Female	*p* value
Anterior ratio	Normal occlusion	78.0 ± 1.8	0.48	78.2 ± 2.6	0.94
	Class I malocclusion	77.0 ± 2.9		78.4 ± 2.3	
	Class II malocclusion	78.0 ± 3.1		78.3 ± 2.5	

Overall ratio	Normal occlusion	91.5 ± 2.1	0.99	91.7 ± 1.8	0.72
	Class I malocclusion	91.6 ± 2.7		91.2 ± 2.4	
	Class II malocclusion	91.5 ± 1.6		91.3 ± 2.3	

**Table 6:** Comparison of Bolton's sample with the present study groups.

	Study group		Bolton's original	*p* value
Anterior ratio	Normal	78.1 ± 2.4	77.2 ± 1.65	0.03^*∗*^
	Class I	77.8 ± 2.7	77.2 ± 1.65	0.11
	Class II	78.2 ± 2.9	77.2 ± 1.65	0.02^*∗*^
	Combined	78.04 ± 2.6	77.2 ± 1.65	0.00^*∗*^

Overall ratio	Normal	91.6 ± 1.9	91.3 ± 1.91	0.31
	Class I	91.3 ± 2.5	91.3 ± 1.91	0.81
	Class II	91.3 ± 2.2	91.3 ± 1.91	0.90
	Combined	91.4 ± 2.2	91.3 ± 1.91	0.50

^*∗*^Significant *p* ≤ 0.05.

**Table 7:** Frequency of distribution of subjects with anterior ratio beyond mean ± 2 SD of Bolton's norms (77.2 ± 1.65).

Group	*N*	Subjects with discrepancy beyond 2 SD		Maxillary excess	Mandibular excess
		Number	%		
Normal occlusion	31	6	19.35	1	5
Class I malocclusion	47	10	21.2	2	8
Class II malocclusion	42	11	26.19	2	9
Total	120	27	22.5	5	22

**Table 8:** Frequency of distribution of subjects with the overall ratio beyond mean ± 2 SD of Bolton's norms (91.3 ± 1.91).

Group	*N*	Subjects with discrepancy beyond 2 SD		Maxillary excess	Mandibular excess
		Number	%		
Normal occlusion	31	2	6.4	2	0
Class I malocclusion	47	6	12.7	2	4
Class II malocclusion	42	3	7.1	2	1
Total	120	11	9.1	6	5

## Data Availability

The datasets used and/or analyzed during the current study are available from the corresponding author on reasonable request.
